# Limited impact of traumatic brain injury on the post-traumatic inflammatory cellular response

**DOI:** 10.1007/s00068-024-02574-z

**Published:** 2024-07-09

**Authors:** F. J.C. van Eerten, E. J. de Fraiture, L. V. Duebel, N. Vrisekoop, K. J.P. van Wessem, L. Koenderman, F. Hietbrink

**Affiliations:** 1 Department of Trauma Surgery, University Medical Center Utrecht, Utrecht, Netherlands; 2 Department of Respiratory Medicine, University Medical Center Utrecht, Utrecht, Netherlands; 3 CTI Center Translational Immunology, University Medical Center Utrecht, Utrecht, Netherlands

**Keywords:** Trauma, Injury, Traumatic brain injury, Neutrophil, Infection, Inflammation

## Abstract

**Purpose:**

Trauma triggers a systemic inflammatory cellular response due to tissue damage, potentially leading to a secondary immune deficiency. Trauma severity is quantified by the Injury Severity Score (ISS). Severe Traumatic Brain Injury (TBI) is associated with high ISSs due to high lethality, despite limited tissue damage. Therefore, ISS might overestimate the post-traumatic inflammatory cellular response. This study investigated the effect of TBI on the occurrence of different systemic neutrophil phenotypes as alternative read-out for systemic inflammation.

**Methods:**

A single-center retrospective cohort study was conducted at a level-1 trauma center. Patients aged *≥* 18 years, admitted between 01-03-2021–01-11-2022 and providing a diagnostic blood sample were included. Four groups were created: isolated TBI, isolated non-TBI, multitrauma TBI and multitrauma non-TBI. Primary outcome was occurrence of different neutrophil phenotypes determined by automated flow cytometry. Secondary outcome was infectious complications.

**Results:**

In total, 404 patients were included. TBI and non-TBI patients demonstrated similar occurrences of different neutrophil phenotypes. However, isolated TBI patients had higher ISSs than their isolated non-TBI controls who suffered similar post-traumatic inflammatory cellular responses. Regardless of the type of injury, patients exhibiting higher systemic inflammation had a high infection risk.

**Conclusion:**

When TBI is involved, ISS tends to be higher compared to similar patients in the absence of TBI. However, TBI patients did not demonstrate an increased inflammatory cellular response compared to non-TBI patients. Therefore, TBI does not add much to the inflammatory cellular response in trauma patients. The degree of the inflammatory response was related to the incidence of infectious complications.

**Supplementary Information:**

The online version contains supplementary material available at 10.1007/s00068-024-02574-z.

## Introduction

It is well recognized that trauma in general induces a post-traumatic inflammatory cellular response [1, 2]. When this response is excessive, it can result in an imbalance in the immune system, which can lead to a secondary acquired immunodeficiency [1, 2]. Consequently, patients who suffer from this imbalance are prone to infections. It has been postulated that patient with severe neurotrauma are predisposed to infectious complications due to several reasons:

1. The post-traumatic inflammatory cellular response is activated by damage associated molecular patterns (DAMPs), which are released from damaged tissues [2–4]. DAMPs trigger the neutrophil compartment of the immunesystem [2–4]. This results in the influx of specific neutrophil phenotype subsets, such as neutrophils with an immature banded nucleus (“left-shift”) and a neutrophil phenotype characterized by a hypersegmented nucleus [2, 4, 5]. Previous research showed that extensiveness of the post-traumatic inflammatory cellular response can be measured based on the presence of banded and hypersegmented neutrophils in the peripheral blood directly after trauma [4].
2. The extent of the post-traumatic inflammatory cellular response is predictive of the development of infectious complications. This is due to a greater risk of imbalance of the immune system [4, 6]. The response increases with the severity of trauma. Severity of trauma is usually expressed by the injury severity score (ISS). Therefore, it has been proposed that ISS correlates to extensiveness of post-traumatic inflammatory response and subsequent risk of infections [4, 6].
3. Traumatic brain injuries (TBIs) are awarded a relatively high ISS compared to other injuries because of their relatively high lethality [7]. It can be hypothesized that TBI patients with high ISSs suffer from a high risk of infection, because it is proposed that ISS correlates to the extent of post-traumatic inflammatory cellular response. In addition, previous research investigated the impact of TBI on the immune system and suggested that neuroinflammation results in immune deficiency and high risk of infections in TBI patients [8–10].

On the other hand, TBI causes less tissue damage relative to trauma of other tissues. As it is hypothesized that the post-traumatic inflammatory cellular response is primarily caused by tissue damage, it might be expected that TBI has less impact on the post-traumatic inflammatory cellular response than trauma of other tissues resulting in a lower risk of infections. This is in contrast to the correlation between high ISS and a high risk of infection as well as the thoroughly investigated neuroinflammation pathway [4, 8–10]. This may result in the observation that ISS does not correlate to the extent of the post-traumatic inflammatory response and subsequent risk of infection in TBI patients.

Our study tested the hypothesis that TBI did not contribute much to modulation of the neutrophil compartment of the innate immune response. In order to investigate this, the effect of TBI on the post-traumatic inflammatory cellular response was investigated using pre-defined neutrophil subset categories [4]. Therefore, the primary aim of this study was to investigate putative differences in neutrophil subset categories comparing trauma patients with and without TBI. The secondary aim of this study was to investigate the relation of the post-traumatic inflammatory cellular response to infectious complications.

## Methods

### Study design

This single-center retrospective cohort study was conducted at the University Medical Center Utrecht (UMCU) between 01-03-2021 and 01-11-2022. All patients included in the study were subjected to neutrophil phenotyping as a standard-of-care procedure following presentation at the trauma bay. Outcomes of flow cytometry were prospectively collected, whereas patient data were retrospectively collected from electronic patient files as well as the Dutch trauma registry. This study was approved by the UMCU ethical review committee (21-1016_AQUIretro2). The processing and storage of data were in accordance with privacy and ethics regulations. The STROBE statement was adhered in order to assure accurate documentation of methods, results, and discussion (appendix 1).

### Patient selection

All trauma patients ≥ 18 years of age admitted to the trauma unit from whom a diagnostic blood sample for determination of neutrophil phenotypes was taken were included. From this existing retrospective, observational cohort database, patients with TBI were identified using data from the trauma registry. There were no exclusion criteria regarding systemic inflammatory responses or metabolic syndromes, as immunosuppressed patients do not show significant differences in numbers of neutrophils subsets as previously described by de Fraiture et al. [4] Lastly, comorbidities, alcohol and drugs abuse were not determined as they are likely missed due to the retrospective design of this study, makingthese unreliable exclusion criteria.

Patients were categorized in four groups: isolated trauma with TBI, isolated trauma without TBI, multitrauma with TBI and multitrauma without TBI. Patients who did not survive or were transferred to another hospital were excluded from the analysis on infectious complications unless they already had developed an infection prior to their transfer or death.

### Patient population definition

*Isolated TBI patients* suffered from an *isolated head* trauma with Abbreviated Injury Scale (AIS) head or skull ≥ 2 and no other AIS > 1. Patients were also included if they had additional facial fractures with AIS > 1 but excluded if they only suffered facial fractures without TBI.

*Isolated non-TBI patients* suffered from an isolated injury of AIS ≥ 2 in only one body region, which was not TBI and no other AIS > 1.

*Multitrauma TBI patients* suffered from both TBI AIS ≥ 2 and ≥ 1 other injury to any other region of AIS ≥ 2, with an ISS ≥ 16 [11]. 

*Multitrauma non-TBI patients* suffered from injuries of AIS ≥ 2 in ≥ 2 body regions, which was not the head or skull, with an ISS ≥ 16 [11]. 

### Data collection

Patient characteristics and outcomes were collected using the trauma registry and supplemented by the patient’s medical file. The following data was collected: age, gender, pre-existing comorbidities, date of trauma, trauma mechanism, type of injury, AIS, ISS, Glasgow Coma Score (GCS) directly after trauma, Intensive Care Unit (ICU) days, Medium Care (MC) days, Length Of hospital Stay (LOS), ventilation days, timing of surgical procedures, in-hospital mortality, and infectious complications. Data on neutrophil surface receptor markers CD16 and CD62L directly after trauma was also collected. Infectious complications were reported in patient’s medical files when they were diagnosed by a clinical practitioner based on clinical signs and laboratory findings (e.g. CRP and leukocyte counts). Imaging or microbiological finds were included in the diagnosis if applicable. Infectious complications were included in this study when the diagnosis was reported in the patient’s medical file in combination with administration of appropriate treatment according to hospital guidelines. Only infections occurring > 48 h after admission and before hospital discharge were included. Moreover, all patients with an expected ICU stay > 48 h received Selective Digestive Decontamination (SDD) in the form of SDD paste consisting of polymyxin B, nystatin and tobramycin. Additionally, intravenous ceftriaxone was administered for 4 days.

### Method of calculating injury severity score

ISS is calculated using AIS. Each individual injury is assigned a body region and a severity scale from 1 to 5. Scale 1 is equivalent to minor injury, 2 to moderate injury, 3 to serious injury, 4 to severe injury, 5 to critical injury and 6 to maximal injury [11–13]. Scoring is dependent on type of injury as well as extensiveness. For example, an extensive traumatic subarachnoid hemorrhage with coma can be scored an AIS 5, yet a minor traumatic subarachnoid hemorrhage without coma can be scored an AIS 2.

### Sample acquisition and AQUIOS CL® flow cytometry

Blood samples were obtained directly after patient arrival at the trauma bay. One 4mL sodium-heparin tube (Greiner Bio-One GmbH, Kremsmünster, Austria) of blood was collected for granulocyte phenotyping. Subsequently, the blood tube was inserted in the AQUIOS CL® “load & go” flow cytometer (Beckman Coulter Life Sciences, Miami, FL, USA) positioned near the shock room. The AQUIOS CL® “load & go” flow cytometer examines the fluorescence of antibody/fluorochrome-combinations specific for different target proteins on the surface of immune cells in the blood, following the protocol described in de Fraiture et al. [4].

### Neutrophil subset categorization

During acute injury induced inflammation, neutrophils can be divided into different phenotype subsets, based on the expression of specific surface proteins (CD16/FcγRIII and CD62L/L-selectin) [4, 14]. Upon acute inflammation two additional phenotypes are found in the peripheral blood. CD16^dim^/CD62L^bright^ neutrophil subsets represent the immature banded phenotype, which are most potent in containing pathogens. CD16^bright^/CD62L^dim^ represent the hypersegmented neutrophil phenotype, which show impaired intracellular bacteria killing and possibly have a more immune regulatory function [14]. 

**Fig. 1:**
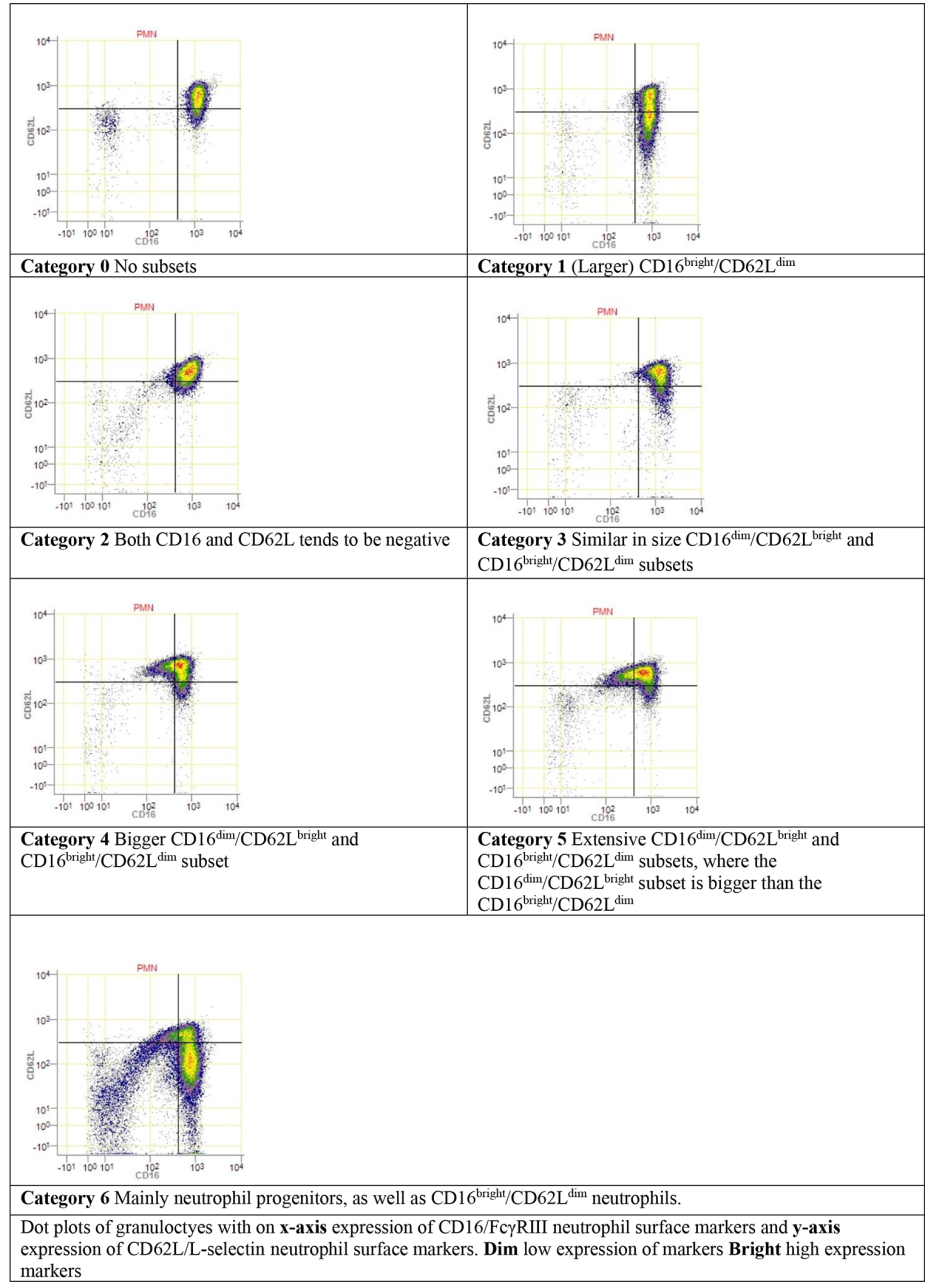
Illustration of expression of neutrophil receptor markers CD16 and CD62L for each category

For each patient, a 2-dimensional CD16/CD62L dot plot was assigned a category 0 to 6 by visual assessment based on the presence of CD16^dim^/CD62L^bright^ and CD16^bright^/CD62L^dim^ neutrophil subsets, as previously described by de Fraiture et al. [4] Fig. 1 shows the different categories. Category 0 shows no subsets, category 1 only shows a CD16^bright^/CD62L^dim^ subset and category 2 shows a subset in the lower left quadrant of the plot which tends to be both CD16 and CD62L negative. Categories 3, 4 and 5 show increasing presence of both CD16^dim^/CD62L^bright^ and CD16^bright^/CD62L^dim^ neutrophil subsets. In category 5, the presence of CD16^dim^/CD62L^bright^ are more distinct than CD16^bright^/CD62L^dim^ neutrophil subsets. Category 6 shows the presence of the CD16^bright^/CD62L^dim^ subset as well as neutrophil progenitors (CD16^dim^/CD62L^dim^). In categories ≥ 3, the categorical number increases with severity of trauma and the concurrent extent of the immune response [4]. The categorization was done by visual assessment by 2 authors. If consensus could not be reached, a third author was consulted.

### Statistical analysis

Statistical analysis was done using R software for statistical computing (version 3.3.2.) [15]. 

Baseline characteristics and outcomes were compared using Mann-Whitney-Wilcoxon test for numerical variables and Chi-square test for categorical variables. Number of TBI and non-TBI patients, their median ISS, and the development of infections in each neutrophil category were compared using Mann-Whitney-Wilcoxon and Chi-square test.

## Results

### Baseline characteristics and outcomes

In total, 404 patients were included who had their neutrophil profile analyzed in the emergency department and were admitted to UMCU. Of these patients, 104 were isolated TBI, 136 isolated non-TBI, 111 multitrauma TBI and 53 multitrauma non-TBI patients.

#### Isolated trauma

Median ISS in the isolated TBI group was 11 (range 4–30) and in the isolated non-TBI group 9 (range 4–26) (*p* < 0.01) (see Table 1). In total, 12% of the isolated TBI patients and 10% of the isolated non-TBI patients developed infections (*p* = 0.78). Median time between hospital admittance and infection development was 8 days (range 3–28) in the isolated TBI group and 6 days (range 2–23) in the isolated non-TBI group. In total, 12% of the isolated TBI patients and 1% of the isolated non-TBI patients did not survive (*p* < 0.01) (Table 2).

**Table 1:** Baseline characteristics of patients with isolated TBI, isolated non-TBI, multitrauma TBI and multitrauma non-TBI

	isolated TBI *n* = 104		isolated non-TBI *n* = 136			multitrauma TBI *n* = 111			multitrauma non-TBI *n* = 53		
					*p*-values						*p*-values
**Median age in years (Range)**	56.5	(18.0–95.0)	44.5	(18.0–93.0)	0.08		55.0	(18.0–93.0)	52.0	(19.0–85.0)	0.24
**Sex n(%)**											
Female	38	(36.5)	27	(19.9)	< 0.01		24	(21.6)	20	(37.7)	0.05
Male	66	(63.5)	109	(80.1)	< 0.01		87	(78.4)	33	(62.3)	0.05
**ASA classification n(%)**											
1	46	(44.2)	65	(47.8)	0.68		53	(47.7)	27	(50.9)	0.83
2	30	(28.8)	52	(38.2)	0.17		33	(29.7)	16	(30.2)	1
3	21	(20.2)	17	(12.5)	0.15		21	(18.9)	10	(18.9)	1
4	3	(2.9)	1	(0.7)	0.44		2	(1.8)	0	(0.0)	0.82
x	4	(3.8)	1	(0.7)	0.22		2	(1.8)	0	(0.0)	0.82
**Immunocompromised n(%)**											
DM1	0	(0.0)	1	(0.7)	1		2	(1.8)	0	(0.0)	0.82
Immunosuppressive medication	1	(1.0)	1	(0.7)	1		2	(1.8)	0	(0.0)	0.82
A splenic	0	(0.0)	0	(0.0)	1		3	(2.7)	2	(3.8)	1
HIV	0	(0.0)	1	(0.7)	1		1	(0.9)	0	(0.0)	1
CVID	0	(0.0)	1	(0.7)	1		0	(0.9)	0	(0.0)	x
**Trauma mechanism n(%)**											
Asphyxia	0	(0.0)	4	(2.9)	0.21		1	(0.9)	2	(3.8)	0.51
Burn incident	0	(0.0)	4	(2.9)	0.21		0	(0.0)	0	(0.0)	x
Drowning	0	(0.0)	0	(0.0)	1		0	(0.0)	0	(0.0)	x
Explosion	0	(0.0)	1	(0.7)	1		0	(0.0)	0	(0.0)	x
Fall	40	(38.5)	36	(26.5)	0.066		37	(33.3)	12	(22.6)	0.22
Hit with blunt object	3	(2.9)	5	(3.7)	1		0	(0.0)	1	(1.9)	0.70
Other	4	(3.8)	16	(11.8)	0.05		5	(4.5)	3	(5.7)	1
Shoot or stabbing incident	0	(0.0)	7	(5.1)	0.05		0	(0.0)	3	(5.7)	0.06
Traffic accident	47	(45.2)	63	(45.3)	0.97		68	(61.3)	32	(60.4)	1
**Median ISS of trauma (Range)**	11	(4–30)	9	(4–26)	< 0.01		26	(16–75)	22	(17–75)	0.44
**Glasgow coma scale n(%)**											
≤ 8	32	(30.8)	7	(5.1)	< 0.01		43	(38.7)	9	(17.0)	< 0.01
> 8	72	(69.2)	128	(94.1)	< 0.01		68	(61.3)	43	(81.1)	0.02
x^*^	0	(0.0)	1	(0.7)	1		0	(0.0)	1	(1.9)	0.70
**Type of injury n(%)**											
Cranial fracture	64	(61.5)	0	(0.0)	< 0.01		68	(61.3)	0	(0.0)	< 0.01
Epidural hemorrhage	8	(7.7)	0	(0.0)	< 0.01		17	(15.3)	0	(0.0)	< 0.01
Subdural hemorrhage	55	(52.9)	0	(0.0)	< 0.01		58	(52.3)	0	(0.0)	< 0.01
Subarachnoid hemorrhage	63	(60.6)	0	(0.0)	< 0.01		59	(53.2)	0	(0.0)	< 0.01
Intracranial hemorrhage	7	(6.7)	0	(0.0)	< 0.01		17	(15.3)	0	(0.0)	< 0.01
Cerebral contusion	44	(42.3)	0	(0.0)	< 0.01		32	(28.8)	0	(0.0)	< 0.01
Light traumatic brain injury	13	(12.5)	0	(0.0)	< 0.01		18	(16.2)	0	(0.0)	< 0.01
Facial fractures	31	(29.8)	11	(8.1)	< 0.01		56	(50.5)	4	(7.5)	< 0.01
Inhalation trauma	0	(0.0)	4	(2.9)	0.21		1	(0.9)	2	(3.8)	0.51
Vascular trauma	0	(0.0)	1	(0.7)	1		7	(6.3)	9	(17.0)	0.06
Submersion	0	(0.0)	1	(0.7)	1		0	(0.0)	0	(0.0)	x
Burn injury	0	(0.0)	1	(0.7)	1		0	(0.0)	0	(0.0)	x
Central cord myelopathy	0	(0.0)	4	(2.9)	0.21		4	(3.6)	9	(17.0)	< 0.01
Vertebral column fracture	0	(0.0)	30	(22.1)	< 0.01		40	(36.0)	24	(45.3)	0.33
Thoracic hollow organ injury	0	(0.0)	21	(15.4)	< 0.01		52	(46.8)	32	(60.4)	0.15
Rib/sternum fracture	0	(0.0)	37	(27.2)	< 0.01		68	(61.3)	38	(71.7)	0.26
Abdomen solid organ injury	0	(0.0)	2	(1.5)	0.60		18	(16.2)	13	(24.5)	0.29
Abdomen hollow organ injury	0	(0.0)	2	(1.5)	0.60		2	(1.8)	5	(9.4)	0.06
Pelvic fracture	0	(0.0)	6	(4.5)	0.09		27	(24.3)	12	(22.6)	1
Peripheral fracture	0	(0.0)	39	(28.7)	< 0.01		63	(56.8)	27	(50.9)	0.60
Crush/traumatic limb amp	0	(0.0)	7	(5.1)	0.05		5	(4.5)	8	(15.1)	0.04

^*^X = no data

**Table 2:** Clinical outcomes of patients with isolated TBI, isolated non-TBI, multitrauma TBI and multitrauma non-TBI

	isolated TBI *n* = 104		isolated non-TBI *n* = 136				multitrauma TBI *n* = 111		multitrauma non-TBI *n* = 53		
					*p*-values						*p*-values
**Admission ward ** ***n*** **(%)**											
Intensive care unit	30	(28.8)	19	(14.0)	< 0.01		50	(45.0)	23	(43.4)	0.98
Intermediate care unit	38	(36.5)	30	(22.1)	0.02		70	(63.1)	36	(67.9)	0.66
Ward	90	(86.5)	122	(89.7)	0.58		96	(86.5)	45	(84.9)	0.97
**Median Length of stay in days (Range)**											
Total	4	(0–85)	4	(0–49)	0.13		8	(0–81)	10	(1–60)	0.26
Intensive care unit	3	(1–26)	2	(1–13)	0.06		4	(1–32)	5	(1–36)	0.45
Intermediate care unit	2	(1–12)	2	(1–7)	0.65		2	(1–32)	3	(1–20)	0.13
Ward	4	(1–81)	4	(1–43)	0.28		7	(1–77)	9	(2–44)	0.09
**Median length of ventilator days**	2	(1–24)	2	(1–13)	0.24		3	(1–14)	3	(1–16)	0.86
Infectious complications n(%)*											
Total patients not transferred or deceased	94	(90.4)	125	(91.9)			91	(82.0)	48	(90.6)	
Total patients with infections	11	(11.7)	12	(9.6)	0.78		15	(16.5)	15	(31.3)	0.07
Total amount of infections	11	(11.7)	13	(10.4)	0.91		15	(16.5)	17	(35.4)	0.03
Type of infection											
Intra-abdominal abscess	0	(0.0)	0	(0.0)	x		0	(0.0)	2	(4.2)	0.22
Intra cranial abscess	1	(1.1)	0	(0.0)	0.89		0	(0.0)	0	(0.0)	x
Line infection	1	(1.1)	0	(0.0)	0.89		0	(0.0)	1	(2.1)	0.74
Meningitis	1	(1.1)	0	(0.0)	0.89		1	(1.1)	0	(0.0)	1
OSM infection	0	(0.0)	0	(0.0)	x		1	(1.1)	1	(2.1)	1
Pneumonia	2	(2.1)	8	(6.4)	0.24		4	(4.4)	7	(14.6)	0.05
Sepsis	0	(0.0)	1	(0.8)	1		1	(1.1)	2	(4.2)	0.57
Soft tissue infection	0	(0.0)	0	(0.0)	x		0	(0.0)	2	(4.2)	0.23
Urinary tract infection	3	(3.2)	2	(1.6)	0.75		5	(5.5)	1	(2.1)	0.62
Viral infection	0	(0.0)	1	(0.8)	1		0	(0.0)	0	(0.0)	x
Wound infection	3	(3.2)	1	(0.8)	0.42		3	(3.3)	1	(2.1)	1
**Median days to first infection (Range)**	8	(3–28)	6	(2–23)	0.34		8	(2–30)	12	(2–37)	0.06
**Mortality n(%)**											
**Total**	12	(11.5)	2	(1.5)	< 0.01		17	(15.3)	6	(11.3)	0.65
**≤ 30 days**	11	(10.6)	2	(1.5)	< 0.01		16	(14.4)	6	(11.3)	0.77

*In total patients who did not decease during hospital admission and/or were transmitted to another hospital, unless they already developed infections. **OSM** osteosynthesis material

#### Multitrauma

Median ISS in the multitrauma TBI group was 26 (range 16–75) and in the multitrauma non-TBI group 22 (range 17–75) (*p* = 0.44) (see Table 1). In total, 17% of the multitrauma TBI patients and 31% of the multitrauma non-TBI patients suffered from infections (*p* = 0.07). Median days to infection was 8 (range 2–30) days in the multitrauma TBI group and 12 (range 2–37) days in the multitrauma non-TBI group. In total, 15% of the multitrauma TBI patients and 11% of the multitrauma non-TBI patients did not survive (*p* = 0.65) (Table 2).

### Distribution of TBI and non-TBI patients among neutrophil categories

The majority of the isolated TBI and isolated non-TBI patients were in category 0: 51% and 52%, respectively (*p* = 0.95) (Table 3). The number of isolated TBI and non-TBI did not differ significantly in the other categories. The majority of the multitrauma TBI and non-TBI patients were in category 3: 34% and 40% respectively (*p* = 0.62). The number of multitrauma TBI and non-TBI patients in each category did not differ significantly.

### TBI and non-TBI patients’ ISS score related to neutrophil categories

**Fig. 2:**
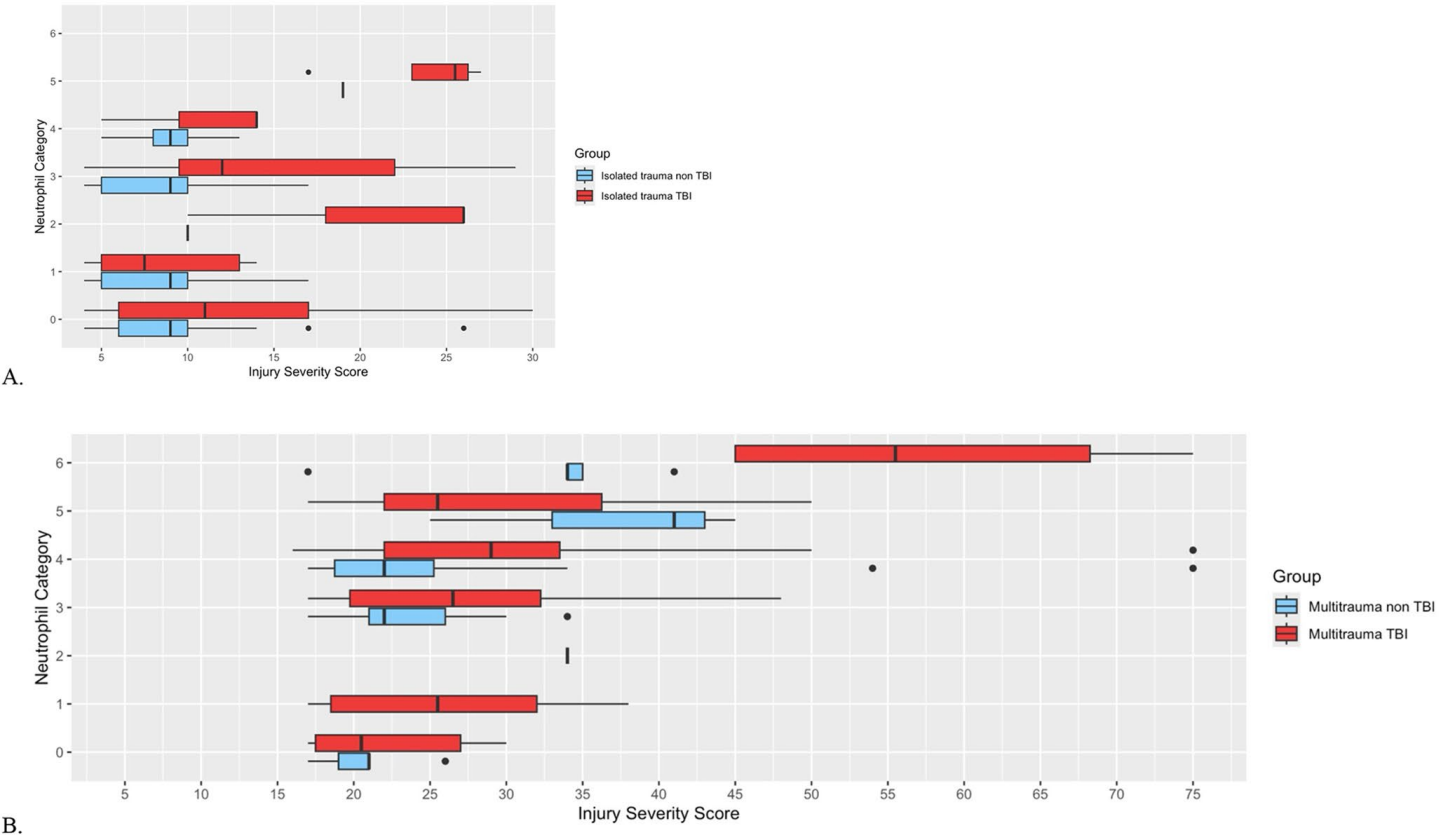
Boxplots ISS versus neutrophil category in TBI and non-TBI patients **A** Isolated trauma patients **B** Multi trauma patients **Y-axis** neutrophil category **X-axis** Median injury severity score. **Blue** Patients with TBI **Red** Patients without TBI

In all four patient groups, the median ISS score increased with the higher neutrophil categories ≥ 3 (Table 4; Fig. 2). Isolated TBI patients had a higher median ISS than isolated non-TBI patients in the categories 0; (*p* < 0.01) and 3 (*p* < 0.01) (Table 4). The multitrauma TBI patients had a higher median ISS than multitrauma non-TBI patients in category 6 (*p* = 0.02) (Table 4; Fig. 2).

**Table 3:** Distribution of patients among the neutrophil categories in TBI patients versus non-TBI patients *n*(%)

**A** Isolated TBI versus isolated non-TBI patients									
		Cat 0	Cat 1	Cat 2	Cat 3	Cat 4	Cat 5	Cat 6	Total
isolated TBI		53 (51.0)	6 (5.8)	3 (2.9)	35 (33.7)	3 (2.9)	4 (3.8)	0 (0.0)	104
isolated non-TBI		71 (52.2)	13 (9.6)	2 (1.5)	36 (26.5)	13 (9.6)	1 (0.7)	0 (0.0)	136
*p*-values		0.95	0.40	0.76	0.29	0.07	0.22	x	
**B** Multi trauma patients with TBI versus multi trauma patients without TBI									
		**Cat 0**	**Cat 1**	**Cat 2**	**Cat 3**	**Cat 4**	**Cat 5**	**Cat 6**	**Total**
multitrauma TBI		22 (19.8)	8 (7.2)	0 (0.0)	38 (34.2)	31 (27.9)	8 (7.2)	4 (3.6)	111
multitrauma non-TBI		5 (9.4)	0 (0.0)	1 (1.9)	21 (39.6)	16 (30.2)	5 (9.4)	5 (9.4)	53
*p*-values		0.15	0.11	0.70	0.62	0.91	0.85	0.24	

Cat = category of neutrophil phenotype subsets

**Table 4:** Median ISS (ranges) per neutrophil category in TBI vs. non TBI patients

**A** Isolated TBI versus isolated non-TBI patients			
	isolated TBI	isolated non-TBI	*p*-values
Cat 5	26 (17–27)	19 (19–19)	0.72
Cat 4	14 (5–14)	9 (5–13)	0.38
Cat 3	12 (5–29)	9 (4–17)	**< 0.01**
Cat 2	26 (10–26)	10 (10–10)	0.32
Cat 1	8 (4–14)	9 (4–17)	0.82
Cat 0	11 (4–30)	9 (4–26)	**< 0.01**
**B** Multitrauma TBI versus multitrauma patients non-TBI			
	**multitrauma TBI**	**multitrauma non-TBI**	***p*** **-values**
Cat 6	56 (45–75)	34 (17–41)	**0.02**
Cat 5	26 (17–50)	41 (25–45)	0.24
Cat 4	29 (16–75)	22 (17–75)	0.15
Cat 3	27 (17–48)	22 (17–34)	0.14
Cat 2	x	34 (34–34)	x
Cat 1	26 (17–38)	x	x
Cat 0	21 (17–30)	21 (17–26)	0.70

**Cat** Category of neutrophil phenotype subsets

### Infections in TBI and non-TBI patients

There were no significant differences in the number of patients with infections in each category between TBI and non-TBI patients, see Table 5. When no distinction was made between TBI, non-TBI, isolated and multitrauma patients, patients in categories 5 and 6 developed significantly more infections than patients in other categories (*p* < 0.01 and *p* < 0.01), see Table 6.

**Table 5:** Number of survivors and non-transferred patients* with infections per neutrophil category in TBI vs. non-TBI patients n(%)

**A** Isolated TBI versus isolated non-TBI patients who suffered from infections (*n*/total)								
	Overall	Cat 0	Cat 1	Cat 2	Cat 3	Cat 4	Cat 5	Cat 6
isolated TBI	11/94 (11.7)	6/49 (12.1)	0/5 (0.0)	1/1 (100.0)	2/29 (6.9)	0/8 (0.0)	2/2 (100.0)	x
isolated non-TBI	12/125 (9.6)	4/67 (6.0)	0/10 (0.0)	0/2 (0.0)	4/32 (12.5)	3/13 (23.1)	1/1 (100.0)	x
*p*-values	**0.78**	**0.73**	**1**	**0.67**	**0.76**	**0.41**	1	x
**B** Multitrauma TBI versus multitrauma non-TBI who suffered from infections (*n*/total)								
	**Overall**	**Cat 0**	**Cat 1**	**Cat 2**	**Cat 3**	**Cat 4**	**Cat 5**	**Cat 6**
multitrauma TBI	15/91 (16.5)	4/20 (20.0)	0/8 (0.0)	x	6/32 (18.8)	3/23 (13.0)	2/7 (28.6)	0/1 (0.0)
multitrauma non-TBI	15/48 (31.3)	1/5 (20.0)	x	0/1 (0.0)	5/21 (23.8)	4/14 (28.6)	2/4 (50.0)	3/3 (100.0)
*p*-values	**0.07**	**1**	**x**	**x**	**0.92**	**0.46**	**0.95**	**0.51**

*Deceased and transferred patients were excluded in this analysis unless they already had developed infections. **Cat** Category of neutrophil phenotype subsets

**Table 6:** Total amount of survivors and non-transferred patients* with infections in both mono-and multi trauma, with and without TBI n(%)

	Cat 0	Cat 1	Cat 2	Cat 3	Cat 4	Cat 5	Cat 6
Infection yes	15 (10.6)	0 (0.0)	1 (25.0)	17 (14.9)	10 (17.2)	7 (50.0)	3 (75.0)
Infection no	126	23	3	97	48	7	1
*p*-values	0.10	0.07	1	1	0.71	**< 0.01**	**< 0.01**

Cat = category of neutrophil phenotype subsets

## Discussion

The results of this study demonstrate that TBI patients having a higher ISS relative to non-TBI patients ended up in the same neutrophil categories that represent the extent of the post-traumatic inflammatory cellular response. This inflammatory cellular response activated through tissue damage was not adequately represented by ISS, especially in the presence of brain injury. TBI causes a high ISS due to high lethality, which results in an over-estimation of ISS regarding the extent of post-traumatic inflammatory cellular response.

It is hypothesized that the post-traumatic inflammatory response is caused by tissue damage as DAMPs trigger the neutrophil compartment [1–4]. As a result banded CD16^dim^/CD62L^bright^ neutrophils and hypersegmented CD16^bright^/CD62L^dim^ neutrophils enter the blood circulation. These neutrophil subsets demonstrate different kinetics in the days following trauma [5]. Banded CD16^dim^/CD62L^bright^ neutrophils are found in the circulation directly after trauma and disappear after 24–48 h [2, 5, 16], whereas hypersegmented CD16^bright^/CD62L^dim^ neutrophils are prominent after 3 days [2, 5, 16]. Interestingly, after 5 days patients become susceptible to infections [2, 5, 14, 16]. This may be caused by exhaustion or depletion of the neutrophil compartment in the bone marrow, as the post-mitotic transfer time is around 5 days implying that upon a high demand, the neutrophil compartment becomes deficient in numbers to deal with infections after 4–5 days [17]. Based on the results of this study it is suggested that TBI causes less tissue damage than other traumatic injuries, which in turn releases less DAMPs to activate the neutrophil compartment. Consequently, a TBI patient with the exact same ISS as a non-TBI patient is less impacted by the post-traumatic inflammatory cellular response.

As mentioned, ISS is a tool to calculate lethality of injuries, which rightfully makes patients with TBI score higher in their ISSs [7, 11–13]. However, the post-traumatic inflammatory cellular response increases with the extent of tissue damage, which does not correlate well with ISS particularly in the case of TBI. Patients who are in the same neutrophil category are believed to suffer from the same extent of tissue damage evoked by hyperinflammation [4]. As TBI leads to relatively high ISSs compared to other trauma with a similar extent of tissue damage, TBI patients will consequently have a higher ISS than non-TBI patients in the same neutrophil category. This means that in TBI patients, ISS poorly correlates to the extent of the post-traumatic inflammatory cellular response and subsequent risk of infections.

Although this current research suggests that ISS overestimates the post-traumatic inflammatory cellular response of the neutrophil compartment in TBI patients, impact of TBI on other parts of the immune system was not investigated here. Different pathways through which TBI affects the immune system have been suggested by previous research. For example, it is proposed that TBI leads to activation of the sympathetic nervous system, which leads to elevation of norepinephrine levels [8, 9]. Catecholamines can inhibit the T-cell function by inhibiting the release of pro-inflammatory cytokines [8, 9]. Moreover, the activation of the hypothalamic-pituitary axis causes release of cortisol, which has immunosuppressive functions, also by inhibiting T-cell function [8, 9]. Following this logic, literature suggests that TBI patients suffer from a relatively high rate of infections [8–10]. In this study there was insufficient power to test the hypotheses that differences can be found when comparing the prevalence of infections in TBI and non-TBI patients. Nevertheless, TBI patients did not show more infections than non-TBI patients in this study. Additionally, it is important to consider that other factors such as ventilation days and number of ICU days influence outcomes such as infections [10]. 

The majority of patients with an isolated injury and a substantial proportion of the multitrauma patients were categorized in neutrophil phenotype category 0. This is in line with previous research on the post-traumatic inflammatory cellular response, where 27% and 23% of trauma patients (a traumageriatric cohort and a pooled cohort of isolated and multitrauma patients, respectively) were categorized in neutrophil phenotype category 0 [4, 18]. This can be explained by the limited tissue damage seen with isolated injury, causing a low post-traumatic inflammatory cellular response. It is therefore not surprising that the majority of these patients were in neutrophil phenotype category 0. The multitrauma patients were mainly in category 3 but still a substantial number were found in category 0, which underlies the potential different influence of different types of injury on the post-traumatic inflammatory cellular response. Due to too low numbers no analysis on the influence of individual injury sites could be performed. In another cohort with severely injured trauma patients which were all administered to the ICU, all patients displayed post-traumatic neutrophil subsets that corresponded with category ≥ 4 [5]. 

Regardless the different types of immune pathways, the post-traumatic inflammatory cellular response of neutrophils as described in this study may potentially be predictive of infectious complications. Clinically, it is valuable to have a tool that can preemptively calculate risk of infectious complications so that clinical precaution can be taken. This research showed that patients in neutrophil subset categories 5 and 6 suffered from significantly more infections than the patients in the other categories, which confirms the findings from previous research [4, 6]. This research also showed that ISS did not directly correlate to the extensiveness of post-traumatic inflammatory cellular response and subsequent risk of infection in the case of TBI. Moreover, ISS is calculated several days after admittance, which is inconvenient in clinical practice. To predict risk of infection directly after trauma, research should be directed towards finding more immediate tools such as changes in neutrophil phenotypes.

A limitation of our study was the absence of the correction for multiple testing in the statistical analysis. Clinical outcomes such as mortality and infections are influenced by many more factors such as ventilation days, days in ICU and comorbidities. Therefore, no causal relation has been found and only an observation of distribution of infectious complications in each neutrophil category was showed. Secondly, the determination of neutrophil categories was performed by authors, which can lead to observer bias. In order to mitigate this issue, the categorization was done by two independent individuals that were blinded for the clinical scores such as injury severity and consensus needed to be reached. Lastly, the conducted study was a retrospective cohort study and consequently, and accuracy of data on infectious complications was dependent on the reporting of the medical personnel, which could also lead to information bias.

## Conclusion

In conclusion, ISS overestimates the extent of post-traumatic inflammatory cellular response, and thus risk of infections in TBI patients. TBI patients did not show enhanced infections nor an increased post-traumatic inflammatory cellular response compared to non-TBI patients. Therefore, ISS overestimated the post-traumatic inflammatory cellular response in TBI patients. Regardless of the type of injury, neutrophil phenotype subset categories directly after trauma did relate to the enhanced incidence of infectious complications.

## Electronic supplementary material

Below is the link to the electronic supplementary material.

Supplementary Material 1: STROBE Statement

## Data Availability

No datasets were generated or analysed during the current study.
